# Supplementary figures and images for: The molecular basis of Abelson kinase regulation by its αI-helix
Source: eLife. 2024 Apr 8;12:RP92324. doi: 10.7554/eLife.92324 (PMC11001296; doi:10.7554/eLife.92324)

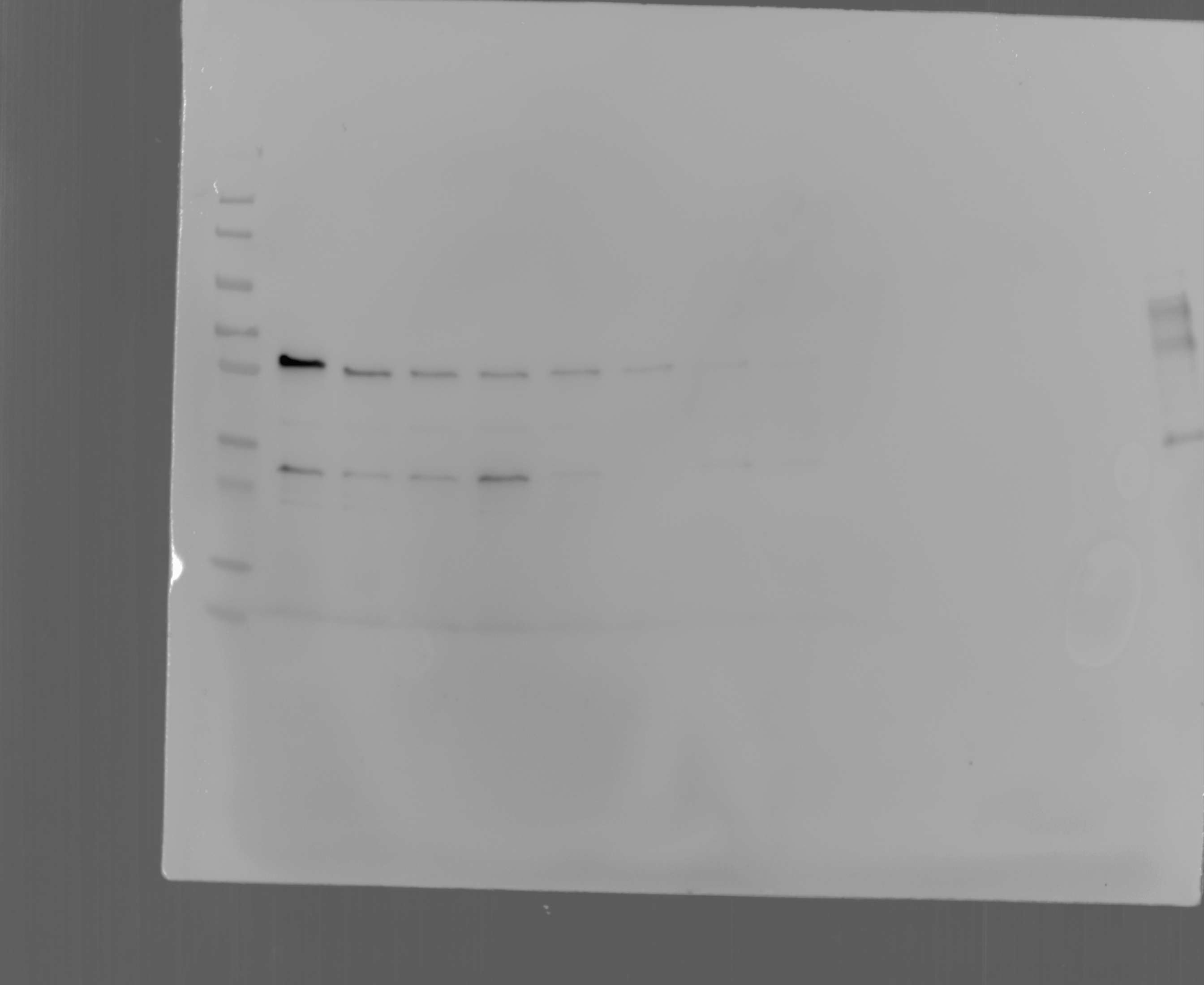

Supplement: Figure 2—source data 1. [file elife-92324-fig2-data1.zip › Figure 2-source data 1/Figure_2_uncropped-gel_original.png]

**B**

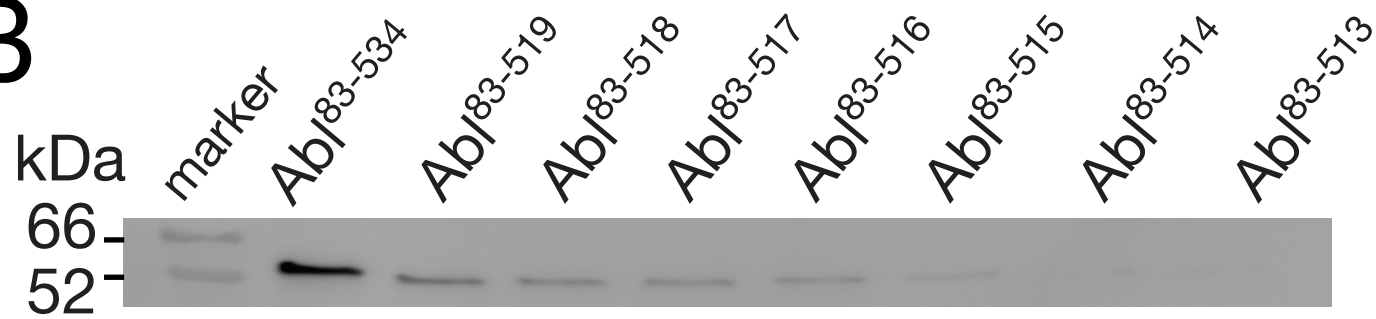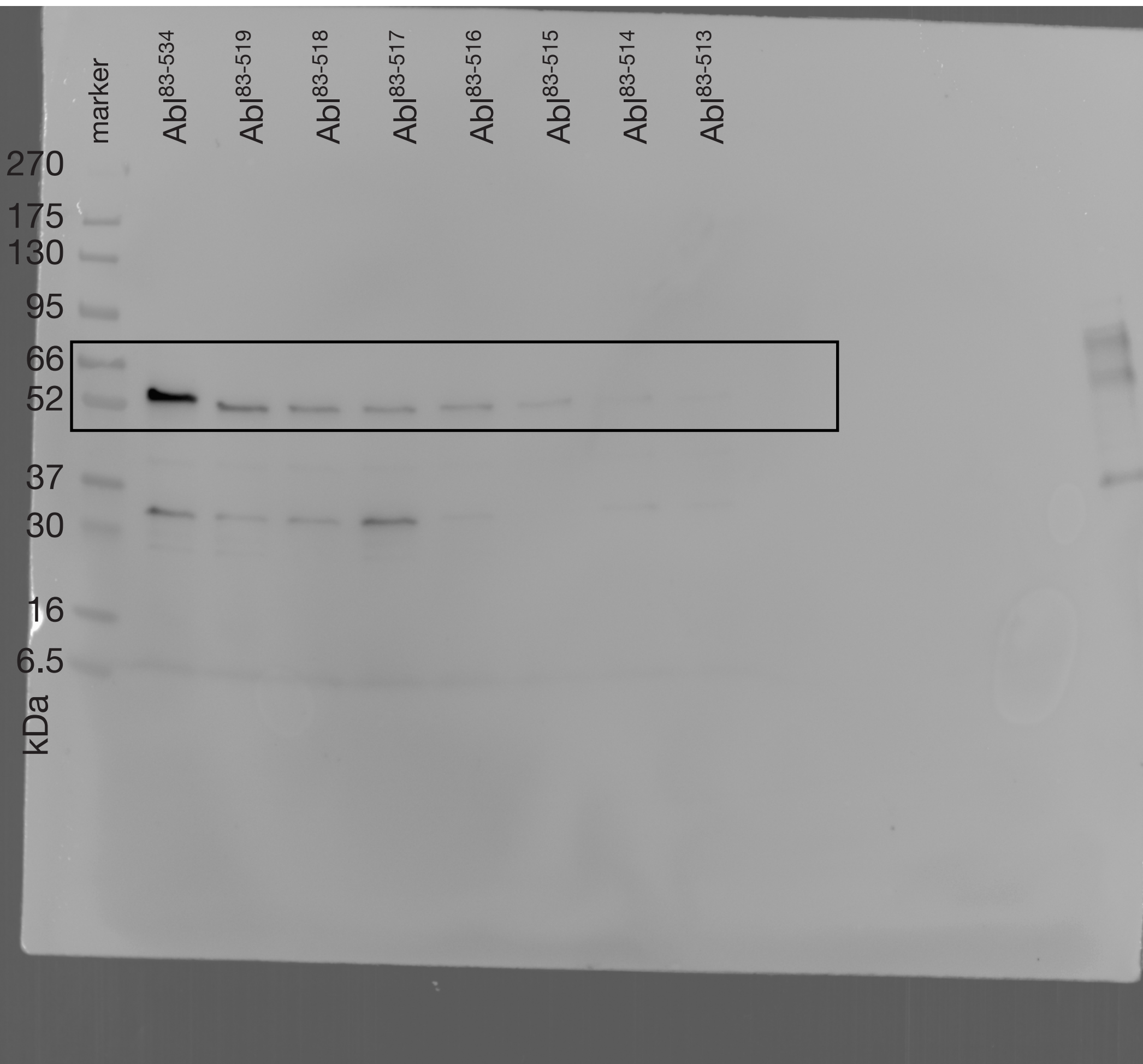

Supplement: Figure 2—source data 2. [file elife-92324-fig2-data2.zip › Figure 2-source data 2/Figure_2_uncropped-gel_annotated.pdf]
